# Effects of Trampling on Morphological and Mechanical Traits of Dryland Shrub Species Do Not Depend on Water Availability

**DOI:** 10.1371/journal.pone.0053021

**Published:** 2013-01-16

**Authors:** Liang Xu, Sofia M. A. Freitas, Fei-Hai Yu, Ming Dong, Niels P. R. Anten, Marinus J. A. Werger

**Affiliations:** 1 College of Life and Environmental Sciences, Hangzhou Normal University, Hangzhou, People’s Republic of China; 2 Ecology and Biodiversity, Institute of Environmental Biology, Utrecht University, Utrecht, The Netherlands; 3 College of Nature Conservation, Beijing Forestry University, Beijing, People’s Republic of China; 4 Centre for Crop Systems Analysis, Wageningen University, Wageningen, The Netherlands; 5 State Key Laboratory of Vegetation and Environmental Change, Institute of Botany, Chinese Academy of Sciences, Beijing, People’s Republic of China; Centro de Investigación y de Estudios Avanzados, Mexico

## Abstract

In semiarid drylands water shortage and trampling by large herbivores are two factors limiting plant growth and distribution. Trampling can strongly affect plant performance, but little is known about responses of morphological and mechanical traits of woody plants to trampling and their possible interaction with water availability. Seedlings of four shrubs (*Caragana intermedia*, *Cynanchum komarovi*, *Hedysarum laeve* and *Hippophae rhamnoides*) common in the semiarid Mu Us Sandland were grown at 4% and 10% soil water content and exposed to either simulated trampling or not. Growth, morphological and mechanical traits were measured. Trampling decreased vertical height and increased basal diameter and stem resistance to bending and rupture (as indicated by the increased minimum bend and break force) in all species. Increasing water availability increased biomass, stem length, basal diameter, leaf thickness and rigidity of stems in all species except *C. komarovii*. However, there were no interactive effects of trampling and water content on any of these traits among species except for minimum bend force and the ratio between stem resistance to rupture and bending. Overall shrub species have a high degree of trampling resistance by morphological and mechanical modifications, and the effects of trampling do not depend on water availability. However, the increasing water availability can also affect trade-off between stem strength and flexibility caused by trampling, which differs among species. Water plays an important role not only in growth but also in trampling adaptation in drylands.

## Introduction

Trampling by large grazers is common in arid and semiarid ecosystems, and may strongly impact plant performance [1]–[3], community structure [4]–[6] and ecosystem functioning [7]. Trampling can directly cause tissue loss and damage and indirectly impose mechanical stress on plants [3], [8]. In arid and semiarid drylands trampling also contributes to desertification [9].

Phenotypic responses of plant growth and morphology to trampling have received increasing attention [2], [3], [10]–[12]. Trampling commonly decreases plants’ height [13]–[15], leaf size [15] and seed size [16]. Also, different species respond differently to trampling [10]–[12], and the differences tend to be associated with growth forms [10], [15], [17]. For instance, erectly growing plant species tend to be less tolerant to trampling than species with a rosette, tussock or otherwise prostrate growth form [10], [11], [17].

However, phenotypic responses of mechanical properties such as stem flexibility, leaf toughness and root strength to trampling have received much less attention, although such responses are likely to contribute to plants’ trampling tolerance [3], [10], [18]. Trampling can impose strong mechanical stress on plants [3], [8], resulting in increased toughness of stems and leaves [10], [17]. Contrary to other forms of mechanical stress such as wind, touch or rubbing that are relatively gentle and well studied, trampling often entails overwhelmingly large forces that plants hardly resist. Stem stress avoidance through increased flexibility is then the only viable response for small or young plants. However, the little work that has been done in this respect has focused mostly on grasses [10]–[12], [14], [15], [17], and we know almost nothing about woody species [3].

In arid and semiarid ecosystems, water is another important factor liming plant performance [19]. It is thus important to determine the extent to which trampling effects on plants are affected by water availability. Increasing water availability can increase allocation to stems, which may increase rigidity, height and diameter that are associated with resistance to trampling, and decrease allocation to roots, which may greatly increase the effects of trampling on the fixation of the root system known as anchorage strength of plants [20]. However, to our knowledge, no study has addressed how water availability can modify effects of trampling on growth, morphology and mechanical traits of plants.

The Mu Us Sandland in north China is a typical semiarid area where overgrazing by large domestic animals is common and desertification is severe [21]. A large number of shrub species are distributed in the Mu Us Sandland, including some relic plants [21]; these shrubs play important roles in protection of local ecosystems. Because of the abundance in shrub species, the Mu Us Sandland is called “kingdom of shrubs” [22]. Four typical shrub species (*Caragana intermedia, Cynanchum komarovi, Hedysarum laeve* and *Hippophae rhamnoides*) of the Mu Us Sandland were selected for the study in order to answer the following questions: (1) What are the effects of simulated trampling on the morphological and mechanical traits of these four species? (2) Do these effects interact with those of water availability, or are they independent of each other? And (3) What are the consequences of the observed shifts in traits for mechanical strength and flexibility at the stem level?

## Materials and Methods

### Study Species

The four species used in this experiment are common and widely distributed in the Mu Us Sandland (37°30′-39°20′N, 107°20′-111°30′E), a semiarid area which forms a main part of the Ordos plateau in northern China [21]. Because of human activity and dry condition of the sandland, degradation of local vegetation and desertification are quite severe [21]. *Caragana intermedia* Kuanget (Leguminosae) and *Hippophae rhamnoides* L. (Elaeagnaceae) are shrubs and *Cynanchum komarovii* Al. (Asclepiadaceae) and *Hedysarum laeve* Maxim. (Leguminosae) are semi-shrubs [23]–[26]. *H. rhamnoides* and *H. laeve* are capable of clonal growth by rhizomes [27], [28]. Except for *C. komarovii*, the other three species are widely used in vegetation recovery and control of sand dunes expansion in this area, palatable to animals and other abilities. *C. komarovii* is poisonous to animals and common in degraded lands, and as important indicator of desertification [23]–[26]. Furthermore, except for *C. komarovii* the other three species can grow to a certainly large size to get rid of threat of trampling. Clonal integration can help clonal plants to resist external impact [3]. Comparing with the adults, the damage of environmental stress on seedlings is much greater.

### The Experiment

Seeds of four species were collected from July to October of 2009 near the Ordos Sandland Ecological Research Station (OSES, 39°29′37.6″N, 110°11′29.4″E, 1300 m a.s.l.) of the Institute of Botany, the Chinese Academy of Sciences, located in the Mu Us Sandland in Inner Mongolia, China. These seeds were from many mother plants distributing in an area of about 3 km^2^, where often suffered from sheep grazing. On 21 December 2009, approximately 400 seeds per species were placed on the surface of shallow plastic square boxes (30 cm×15 cm×5 cm, 2.25 L) filled with sand and underwent a stratification period of two weeks at a temperature of 5°C. In January 2010, germinated plants were transplanted to small square pots (7 cm×7 cm×8 cm, 0.24 L). After another three months of growth, seedlings of similar sizes were transplanted to 6.5 L pots (24 cm in diameter and 21 cm in height) fully filled with sand. Extra two grams solid fertilizer (16N-11P_2_O_5_-11K_2_O-3MgO+Te, 3–4 months, Osmocote Exact, Scotts International B.V, Heerlen, the Netherlands) were added to each pot and mixed evenly with sand.

On 13 May 2010, the seedlings of each species were randomly subjected to one of four combined treatments including two levels of soil water availability (4% *vs.* 10% water content for dry and wet conditions, see File S1) and two levels of trampling (with *vs.* without). There were 13 replications of each of the four conditions (water and trampling, low and high) for three of the four plant species, and 12 replications for *C. intermedia*. During the experiment the volumetric soil water content was maintained nearly constant at 4% or 10% by periodical measurement with a Time Domain Reflectometer (Wageningen, the Netherlands). Trampling was applied with a manually constructed load whose weight (6.22 kg) and surface area (3.07 cm in diameter) were such that, when placed on a plant, it produced a stress level (0.84 kg/cm^2^) similar to that produced by the hoof of a 40 kg sheep [29]. The load consisted of one long metal pipe and four short metal pipes. The short pipes were fixed tightly with tape and rope to the middle of the long pipe. Pipes were filled with concrete to obtain the appropriate weight. After that pipes were sealed well with tape to avoid leakage. A cap with the diameter of 3.07 cm was installed at the bottom of the long pipe. In the ecosystem where we concern, the grazing is mainly in a free way without specific location. Trampling was simplified and applied three times a week for 10 second each time. The trampled direction was changed each time. Positions of pots were altered every two weeks to avoid block effects.

The experiment was carried out in a climate-controlled greenhouse compartment located at the Utrecht University Botanical Gardens, the Netherlands (52° 5' 16.79''N, 5° 10' 8.26'E). The average temperature was 18°C and the average humidity 40%.

### Measurements

On 20 June 2010, all experimental plants were harvested. Stem length, vertical height from soil surface to the top of trampled plants, basal diameter and leaf thickness of main shoots were measured. Main shoots of plants were separated into leaves, stems and roots. Leaf area was measured with a LI-3100 leaf area meter (LI-COR, Inc. Lincoln, Nebraska, USA). Aboveground shoots were packed in wet tissue to avoid turgor loss and taken to the lab to measure mechanical traits. Dry mass of different organs were determined after drying at 70°C for 48 h. Small new shoots generated during the treatment period were not conducted as the main. We only measured the whole dry mass of the new shoots, including leaves and stems.

We determined four mechanical traits: Young’s modulus (*E*), breaking stress (*σ_b_*), yield stress (*σ_y_*) and maximum load force of main stems using a universal electromechanical testing machine (Type 5542, Instron, Norwood, Massachusetts, USA) and applying the three-point bending technique (for details see [30]). Vertically applied forces (*F*; N) and resulting deflections (δ; m) were recorded. The distance between supports was adjusted such that it was always approximately 15 times the diameter of the stem section.

We calculated leaf mass area (LMA, g cm^−2^) and leaf mass density (LMD, g cm^−3^) to describe the leaf characteristics. To present the mechanical traits of plants’ stems, a number of parameters were measured and calculated, including: the second moment of area (*I*, m^4^) describing the geometric contribution to stiffness of the stem [31], [32], the Young’s modulus (*E*) representing stiffness of an elastic material [33], [34], flexural stiffness (*EI*, N m^−2^) of the rigidity of a stem cross section, the stem yield stress (*σ*_y_) when stems started bend and the break stress (*σ*_b_) when stems became broken, which quantifies the resistance of stem tissue to bending and rupture. Details of calculations of these mechanical traits can be found in [33].

The stem traits described above were used to calculate a number of parameters that indicate whole-stem behavior under externally applied forces. We note that these are qualitative measures of stem mechanical behavior solely used for comparative purposes. We calculated the minimum bending force (*F*_bend_) referring to bending the stem and the minimum lateral break force (*F*_break_) required to rupture the stem at its base. In this study we assume that trampling exerts an overwhelmingly large force on plants and consider a favorable balance between stem strength to bending resistance to reduce the chance of damage. We use *F*_break/_ *F*_bend_ as a simple proxy to describe this balance, which shows that the balance between strength and flexibility is related to break stress, shoot vertical height, Young’s modulus and basal diameter (details in [3]).

### Statistical Analysis

A three-way ANOVA was used to test the effects of species, trampling and water availability on growth, morphological and mechanical traits. Before analysis, data were checked for equality of variance with Levene’s test and for normality with Shapiro-Wilk’s test. SPSS 16.0 (SPSS Inc., Chicago, Illinois, USA) was employed for the analyses. Here we chose *P*<0.05 as significance level.

## Results

### Growth and Morphological Traits

**Figure 1:**
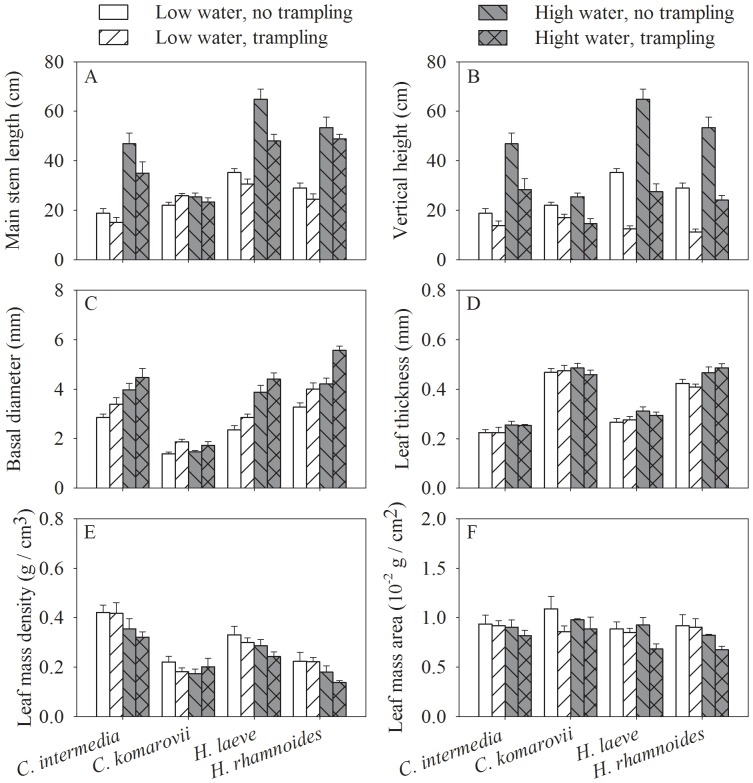
Effects of water availability and trampling on leaf traits and stem traits of the four species. The panel A, B, C, D, E and F are for main stem length, vertical height, basal diameter, leaf thickness, leaf mass density and leaf mass area respectively. Data are mean ± SE without transformation.

Trampling significantly reduced the main stem length in all species except of *C. komarovii* (Table 1; Fig. 1A) and caused stems to be less inclined, thus reducing the distance between soil level and stem apex (i.e., vertical height), albeit less strongly in *C. intermedia* and *C. komarovii* than in the other two species (Table 1; Fig. 1B). Trampling increased consistently stem diameter (Table 1; Fig. 1C). Plants under high water availability had larger stem length, vertical height and diameter in all species except *C. komarovii* (Table 1; Fig. 1A, B, C). Trampling did not significantly affect leaf thickness (Table 1; Fig. 1D) or leaf mass density (LMD; Fig. 1E), but did significantly decrease leaf mass area (LMA; Fig. 1F). Increasing water availability increased leaf thickness (Table 1; Fig. 1D) and decreased LMD (Fig. 1E) and LMA (Fig. 1F) of all four species except *C. komarovii*.

**Table 1:** Results of ANOVA for the effects of species (S), trampling (T), water (W) and their interactions on morphological and growth properties of four species.

Effect	Stem length	Vertical height	Diameter	Leaf thickness	LMD	LMA	Biomass	Leaf ratio	Stem ratio	Root ratio	New shoot ratio	df
Species	69.82^***^	14.01^***^	194.68^***^	194.86^***^	45.40^***^	1.89^ns^	61.58^***^	84.73^***^	31.08^***^	84.43^***^	51.35^***^	3
Trampling	13.91^***^	164.12^***^	37.77^***^	0.15^ns^	2.42^ns^	6.24^*^	2.72^ns^	1.61^ns^	0.51^ns^	0.64^ns^	0.02^ns^	1
Water	181.96^***^	95.44^***^	64.17^***^	14.16^***^	15.67^***^	6.17^*^	94.82^***^	324.32^***^	19.29^***^	440.31^***^	80.88^***^	1
S×T	3.22^*^	9.59^***^	0.59^ns^	0.10^ns^	0.22^ns^	0.44^ns^	2.70^*^	1.89^ns^	3.76^*^	0.88^ns^	1.86^ns^	3
S×W	20.74^***^	13.84^***^	10.63^***^	2.30^ns^	0.96^ns^	0.36^ns^	18.41^***^	21.58^***^	6.26^***^	28.76^***^	25.25^***^	3
T×W	3.85^ns^	1.15^ns^	0.53^ns^	0.18^ns^	0.03^ns^	1.15^ns^	2.41^ns^	3.50^ns^	1.88^ns^	0.03^ns^	0.32^ns^	1
S×T×W	1.82^ns^	2.94^*^	0.87^ns^	0.89^ns^	0.89^ns^	1.03^ns^	2.66^ns^	0.43^ns^	0.60^ns^	0.57^ns^	0.38^ns^	3

*F* values and significance levels (^***^ *P*<0.001, ^**^ *P*<0.01, ^*^ *P*<0.05 and ^ns^ *P*≥0.05) are given. Data were Ln-transformed before analyses.

**Figure 2:**
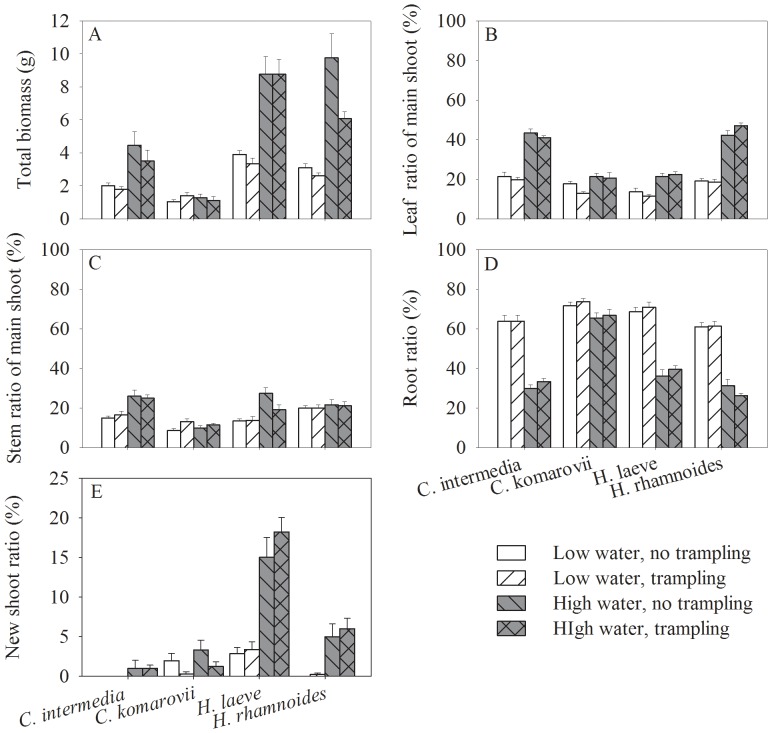
Effects of water availability and trampling on biomass and allocation of the four species. The panel A, B, C, D and E are for total biomass, leaf mass ratio of main shoot, stem mass ratio of main shoot, root ratio and new shoot ratio respectively. Data are mean ± SE without transformation.

Trampling decreased biomass of *H. rhamnoides* but had no significant effect in the other three species (Table 1; Fig. 2A). The fractional biomass allocation to different plant parts was not significantly affected by trampling, except in *C. komarovii* where stem allocation increased (Table 1; Fig. 2B, C, D, E). Increasing water content increased final biomass in all species except *C. komarovii* (Table 1; Fig. 2A). Under low water content, more biomass was allocated to roots in all four species, at the expense of allocation to other organs (Table 1; Fig. 2B, C, D, E).

### Mechanical Properties

**Figure 3:**
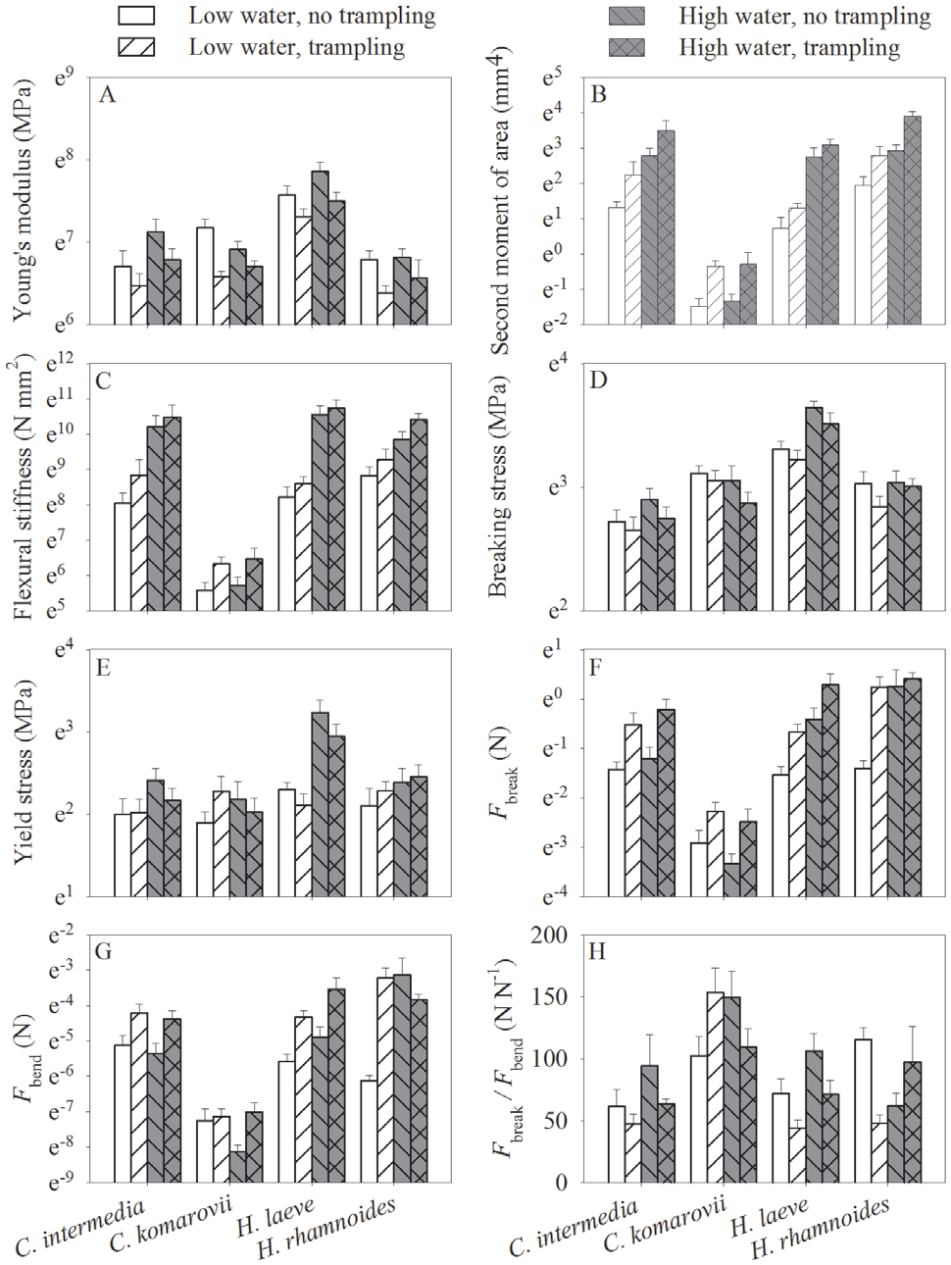
Effects of water availability and trampling on mechanical traits of the four species. The panel A, B, C, D, E, F, G and H are for Young’s modulus, second moment of area, flexural stiffness, break stress, yield stress, minimum lateral break force (*F*_break_), bend force (*F*_bend_) and *F_break/_F_bend_* respectively. Data are mean ± SE. Except for h, values of y axis are Ln-transformed as a result of low values of *C. komarovii*.

Trampling reduced tissue rigidity in terms of the Young’s modulus (*E*) for all four species, under both dry and wet conditions (Table 2; Fig. 3A). However, trampling increased basal diameter (Table 1; Fig. 1C) which strongly determines the second moment of area (*I*; Table 2; Fig. 3B). As the product of *E* and *I*, flexural stiffness (*EI*) exhibited a similar trend as *I* (Table 2; Fig. 3C), indicating that variation in *I* contributed more to flexural stiffness than variation in *E*. Compared with the other three species, stems of *C. komarovii* had very low flexural stiffness, which could be attributed to the thinner stems with a lower *I*. Trampling also resulted in a consistent but small reduction in the break stress (*σ_b_*), but had no significant effect on the yield stress (*σ_y_*; Table 2; Fig. 3D, E). In *H. laeve* at high water availability had a strong positive effect on *σ_b_* and *σ_y_* values. The effect of soil water content on tissue properties clearly differed between species (Table 2). Low water content reduced *E* in *C. intermedia* and *H. leave*, but not in the other two species. Similarly it reduced *σ_b_* and *σ_y_* only in *H. laeve*.

**Table 2:** Results of ANOVA for the effects of species (S), trampling (T), water (W) and their interactions on mechanical properties of four species.

Effect	*E*	*I*	*EI*	*σ_b_*	*σ_y_*	*F_break_*	*F_bend_*	*F_breakl/_F_bend_*	df
Species	40.00^***^	167.46^***^	117.89^***^	36.18^**^	11.40^***^	54.87^***^	56.25^***^	13.29^***^	3
Trampling	22.04^***^	19.33^***^	9.02^**^	5.05^*^	0.28^ns^	29.26^***^	31.31^***^	6.82^**^	1
Water	4.21^*^	63.84^***^	54.75^***^	5.70^*^	16.57^***^	4.43^*^	0.61^ns^	4.98^*^	1
S×T	0.31^ns^	0.66^ns^	0.07^ns^	0.07^ns^	1.14^ns^	0.15^ns^	0.57^ns^	2.13^ns^	3
S×W	2.11^ns^	11.44^***^	8.14^***^	4.84^**^	4.61^**^	4.75^**^	2.76^*^	2.47^ns^	3
T×W	0.05^ns^	0.01^ns^	0.17^ns^	0.04^ns^	1.98^ns^	0.03^ns^	0.55^ns^	0.94^ns^	1
S×T×W	0.91^ns^	1.16^ns^	0.40^ns^	0.41^ns^	0.16^ns^	1.03^ns^	3.98^*^	7.39^***^	3

*F* values and the significance levels (^***^ *P*<0.001, ^**^ *P*<0.01, ^*^ *P*<0.05 and ^ns^ *P*≥0.05) are given. Data were Ln-transformed before analyses.

To determine stem resistance to either breakage or bending we calculated the minimum forces required to either rupture the stem at its base (*F*_break_) or start to bend it (*F*_bend_). *F*_break_ thus indicates stem strength and *F*_bend_ resistance to bending. Trampling increased both *F*_break_ and *F*_bend_ across all species and both water treatments (Table 2; Fig. 3F, G). The ratio *F*_break*/*_ *F*_bend_, proxy for the balance between strength and flexibility, of *C. intermedia* and *H. laeve* was decreased by trampling (Table 2; Fig. 3H). Trampling enhanced the ratio in *C. komarovii* under low water content but reduced under in high water content and with the opposite in *H. rhamnoides* (Table 2; Fig. 3H).

## Discussion

Most studies on trampling were conducted at the community level [35]–[37], and few focused on the plastic responses of individual plants. However, in mobile or semi-fixed dunes in drylands where plants tend to grow solitarily and thus vegetation is sparse [38], studying responses of individual plants to trampling is more practical. The trampling treatment used in our study simulated a relatively frequent impact of sheep, the most common grazer in the Mu Us Sandland. We found that trampling had no effect on the survival of the seedlings of all the four species, and had little effect on final biomass, suggesting that juveniles of these woody species are tolerant to trampling.

Trampling decreased stem length and vertical height of the four shrub species, agreeing with previous findings on herbaceous species [13]–[15] and suggesting that sensitivity of height to trampling is common responses to trampling. Under trampling, plants may generate various other changes in morphological and mechanical traits on the whole-plant level, including decrease in leaf size [15] and seed size [16] and increase in stem and leaf toughness [10], [17]. Relatively small leaf may be affected less by trampling. Increased toughness can help leaves to resist the impact. However, our results of leaf characteristics are different from others, very likely because trampling was directly imposed on stem in our study, but on the whole plant in other studies [10], [15]. Reduction in stem elongation (except of *C. komarovii*) and declination by trampling together resulted in diminishing the height of the stem apex from the sand. Trampling also increased radial stem growth. Thicker stem can support more self-load and bear more external impact. Therefore variances in diameter may contribute to a larger flexural rigidity and a larger resistance to bending and rupture.

In the Mu Us Sandland, the rainfall is in uneven inter- and intra-annual heterogeneity [19]. Generally the precipitation is mainly concentrated from July to September [21]. The summer time is thus important for germination of seeds and growth of seedlings. Rapid growth in the growing season can help the small seedlings to resist the external disturbance. Contrary to our expectation, the effects of trampling on the mechanical traits of the four shrub species did not depend on water availability. One of the main effects of trampling as mechanical stress is to decrease rigidity of stem (Young's modulus, *E*). In a previous study, water availability also did not affect the effects of shaking (another type of mechanical stress) on the stem rigidity of *H. laeve* [38]. However, for the annual herb *Corispermum mongolicum*, it was found that shaking reduced its stem tissue rigidity at low water availability but increased it at high water availability [39]. Therefore, it seems that responses of plants to mechanical stress depend on the plant type (shrubs *vs*. herbs) and strength of the stimulation (one time of overwhelming stress–trampling *vs*. other type. Turgor pressure plays a more important role in determining stem rigidity in herbaceous plants than in woody plants [40]. In woody plants stem mechanical traits are less sensitive to their water status. Mechanical responses of woody species to mechanical stress are likely to depend mainly on composition and quantity of tissue (e.g. parenchyma, sclerenchyma, xylem, vascular tissue, and so on) but not the turgor pressure. However, plant resistance to external forces relies on both mechanical and morphological traits at the whole stem level. Though increasing water availability had no interactive effect with trampling on stem rigidity, it did increase basal diameter and associated geometric stiffness which allowed more external impact. Nevertheless, the response of herb *Corispermum mongolicum* may be the species-specific reaction. More species and work are needed to test the responses of woody plants and herbs to the interaction of mechanical stress and water availability.

Plants can prevent mechanical damage by building a flexible stem that easily reconfigures to minimize the amount of force encountered (stress avoidance), by building strong structures that resist large forces (stress tolerance), or by both [41], [42]. A trade-off between strength and flexibility can be expected because thicker structures are stronger but less flexible than thinner structures [41]. Such a trade-off inevitably imposes constraints on the expression of traits associated with both strategies. Because trampling of small plants by large mammals represents an overwhelmingly large force, stress avoidance is most likely the viable strategy to prevent damage. However, both trampling and increasing water availability resulted in thicker stem to increase the tolerance. In that case, plants need to build a favorable balance between avoidance and tolerance. In the present study stress avoidance was characterized by the inverse of minimum bend force (1/*F*_bend_), stress tolerance by the minimum break force (*F*_break_) and the balance by the ratio of break and bend force (*F*_break/_ *F*_bend_). Overall, we found that trampling enhanced both *F*_break_ and *F*_bend_, meaning more force are needed to bend or break trampled shoots. The trampling induced increase in stem diameter and associated increases in *F*_break_ and *F*_bend_ are consistent with other researches about responses of plants to other forms of mechanical stress (e.g. wind, rubbing and flexing; [33], [43], [44]). These latter forms of mechanical stress usually entail smaller forces than trampling. In semi-arid regions wind is the predominant form of mechanical stress and it is possible that thigmomorphogenic responses in our species act to reduce wind damage rather than trampling damage.

On the other hand, generally trampling increased *F*_bend_ more, thus reduced values of *F*_break/_ *F*_bend_, reflecting a less favorable balance between strength and flexibility. The exception is *C. komarovii* under the low water availability and *H. rhammoides* under the high water availability, where trampling increased *F*_break/_ *F*_bend_. As argued in the previous paragraph, this enhanced strength at the expense of flexibility in response to trampling seems maladaptive as trampling represents an overwhelming force. On the other hand the observed reduction in stem inclination angle may help reduce the chances of mechanical damage under trampling as the bending required to press the plant shoot to the soil becomes smaller.

In drylands, resources are highly heterogeneously distributed [45], which possibly leads to a large range of variance in phenotypic plasticity, especially combined with other external stresses. In this study we find that trampling significantly affected the morphological and mechanical traits associated with the stem resistance to bending and rupture in all four species. The results also show that not only in growth, but also in trampling resistance, water played an important role in drylands. Furthermore, the effects of water on trade-off between stem strength and flexibility caused by trampling differed among species. However, there was no significant interaction between mechanical disturbance and water availability among species. Comparing with other studies [38], [39], it is likely that interaction is probably more common in herbaceous than in woody species. More work is needed to investigate the potentially different interactions between water and mechanical stress among species.

## Supporting Information

File S1 Soil Water Characteristic.
(DOC)
