# Supplementary material for: Effects of Trampling on Morphological and Mechanical Traits of Dryland Shrub Species Do Not Depend on Water Availability
Source: PLoS One. 2013 Jan 16;8(1):e53021. doi: 10.1371/journal.pone.0053021 (PMC3547011; doi:10.1371/journal.pone.0053021)
Supplement: File S1 — Soil Water Characteristic. (DOC) [file pone.0053021.s001.doc]

**Soil Water Characteristic**

Our intention was to implement drought treatments in such a way that plants of different species and trampling treatments would experience very similar soil water potentials. Water potential is the energy associated with the forces to hold the water in the soil. For each particular soil there is a unique relationship between water content and water potential. Water potential is the standard to define the dry and wet condition while water content is easily monitored to reflect the water potential. Therefore, a small experiment was conducted to measure the relationship between the water potential and water content of sand to determine the levels of water treatments. Five square pots (13 cm×13 cm×13 cm, 1.55L) were filled with dry sand which would be used in the following experiment. Sand was dried at 105℃ in the stove for one days. One gypsum block was inserted into each pot at 10 cm connecting to the soil moisture measuring system (Eijkelkamp, Giesbeek, the Netherlands). Weights of pots filled with dry sand and the gypsum block were measured before the measurement. As the pots for experiment were not very large, we considered sand of the whole pots had the same water potential. Every hour, 30 ml water was added to the pot. The machine recorded the soil water potential while the water content of the soil mixture was gravimetrically (*θ_g_*) estimated using the following equation:

(1)

where *m_wet_* is weight of moisture soil and *m_dry_* is weight of the same soil sample which was dried for two days at 105°C until weight became constant.

The volumetric water content (*θ_v_*) was estimated as:

(2)

where density of sand (ρ_sand_) was measured with the dried sand, and density of water was treated as 1g / cm^3^.

According to the water characteristic curve of volumetric water content and water potential (Fig. 1) and other study (Martine *et al.* 2008), the values of 4% and 10% volumetric water content were chosen and correspond respectively to soil water potentials of -13 Bars (low soil water condition) and -2 Bars (high soil water condition).

**Fig. 1** Soil water characteristic describes the relationship between volumetric soil water content (%) and soil water potential (Bar). The dots are fitted and showed as y = 12.921*x ^(-0.4579)^ (*R*^2^=0.9012).
